# Comparative, transcriptome analysis of self-organizing optic tissues

**DOI:** 10.1038/sdata.2015.30

**Published:** 2015-06-23

**Authors:** Munazah Andrabi, Shigehiro Kuraku, Nozomu Takata, Yoshiki Sasai, Nick R. Love

**Affiliations:** 1 Phyloinformatics Unit, RIKEN: Center for Life Science Technologies, Kobe 650-0047, Japan; 2 Organogenesis and Neurogenesis Group, RIKEN: Center for Developmental Biology, Kobe 650-0047, Japan; 3 Laboratory for In Vitro Histogenesis, RIKEN: Center for Developmental Biology, Kobe 650-0047, Japan

**Keywords:** Stem-cell differentiation, RNA sequencing, Retina, Developmental neurogenesis

## Abstract

Embryonic stem (ES) cells have a remarkable capacity to self-organize complex, multi-layered optic cups *in vitro* via a culture technique called SFEBq. During both SFEBq and *in vivo* optic cup development, Rax (Rx) expressing neural retina epithelial (NRE) tissues utilize Fgf and Wnt/β-catenin signalling pathways to differentiate into neural retina (NR) and retinal-pigmented epithelial (RPE) tissues, respectively. How these signaling pathways affect gene expression during optic tissue formation has remained largely unknown, especially at the transcriptome scale. Here, we address this question using RNA-Seq. We generated Rx+ optic tissue using SFEBq, exposed these tissues to either Fgf or Wnt/β-catenin stimulation, and assayed their gene expression across multiple time points using RNA-Seq. This comparative dataset will help elucidate how Fgf and Wnt/β-catenin signaling affect gene expression during optic tissue differentiation and will help inform future efforts to optimize *in vitro* optic tissue culture technology.

## Background & Summary

**Figure 1:**
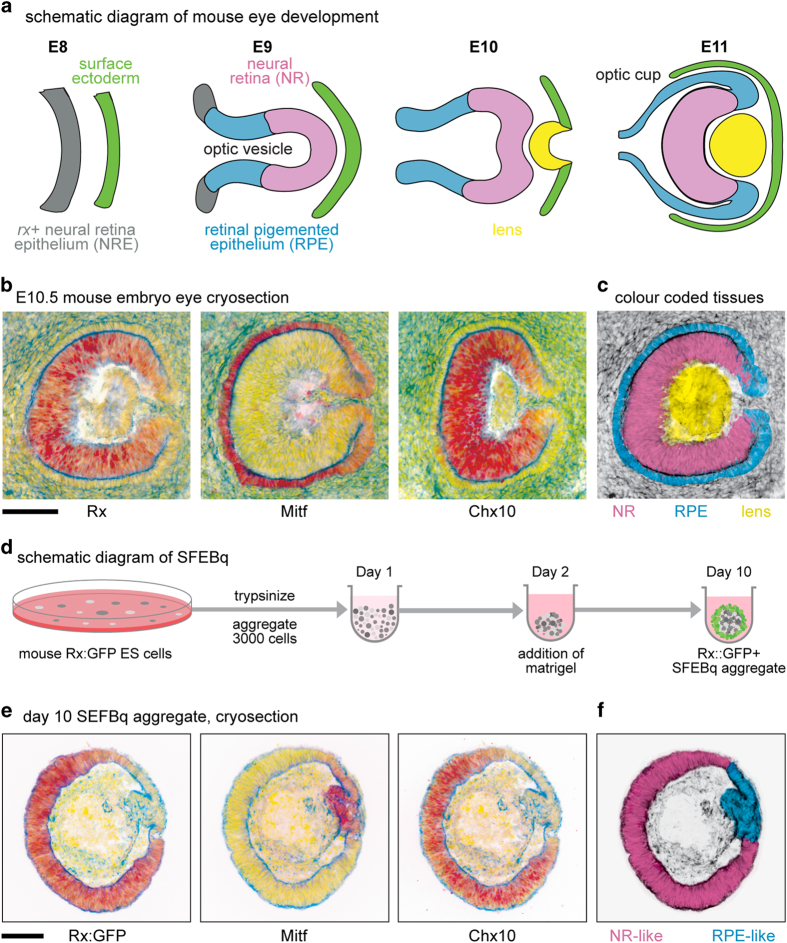
Optic cup development, gene expression, and schematic diagram of SFEBq culture. (**a**) Schematic diagram of murine eye development from embryonic days E8—E11, a time in which the neural retina (NR) and retinal pigmented epithelial (RPE) tissues emerge. (**b**) Cryosections of an E10.5 mouse embryo underwent immunohistochemistry, selected gene expression displayed in red, DAPI shown in yellow, Actin shown in blue. Scale bar 100 μm. (**c**) Pseudocoloured E10.5 cryosection showing developing NR, RPE and lens. (**d**) Schematic diagram of SFEBq, a technique that generates ES-cell aggregates with a peripheral layer of Rx+ optic progenitor tissue. (**e**) Cryosections of Day 10 SFEBq Rx:GFP aggregate with a continuous peripheral layer of optic progenitor tissue underwent immunohistochemistry. Marker gene expression displayed in red, DAPI shown in yellow, Actin shown in blue. Scale bar 100 μm. (**f**) Pseudocoloured diagram of a Day 10 SFEBq aggregate with differentiating NR-like and RPE-like tissues.

During development, *rx* expressing neural retina epithelium (NRE) differentiates into neural retina (NR) and retinal pigmented epithelium (RPE)^1^ (Fig. 1a), tissues with distinct morphologies and gene expression patterns (Fig. 1b,c). For instance, NR tissues show a comparatively thickened morphology, express the transcription factor gene *chx10* (also called *vsx2*), sustain a high expression level of *rx*, and eventually perform the light-sensing duties of the mature eye^2^ (Fig. 1b). Conversely, the RPE is a comparatively thin, pigmented monolayered epithelium that expresses the transcription factor Mitf^1^ (Fig. 1b) and other pigmented cell markers such as tyrosinase (*tyr*) (note, Mitf is also expressed in pigmented cells such as melanophores and peripheral retinal components such as the pigmented ciliary body^3^).

Amazingly, embryonic stem cells have the capacity to recapitulate optic cup formation via a culture technique called SFEBq (serum-free floating culture of embryoid body-like aggregate with quick reaggregation)^4–7^ (Fig. 1d, Supplementary Fig. 1a). SFEBq-generated optic cups, although not totally analogous to their *in vivo* counterparts, facilitate the self-organization of Chx10+ NR-like and Mitf+ RPE-like tissues^7^ (Fig. 1e,f). In this way, SFEBq provides a convenient *in vitro* method to generate NR-like and RPE-like tissue for further analyses.

**Figure 2:**
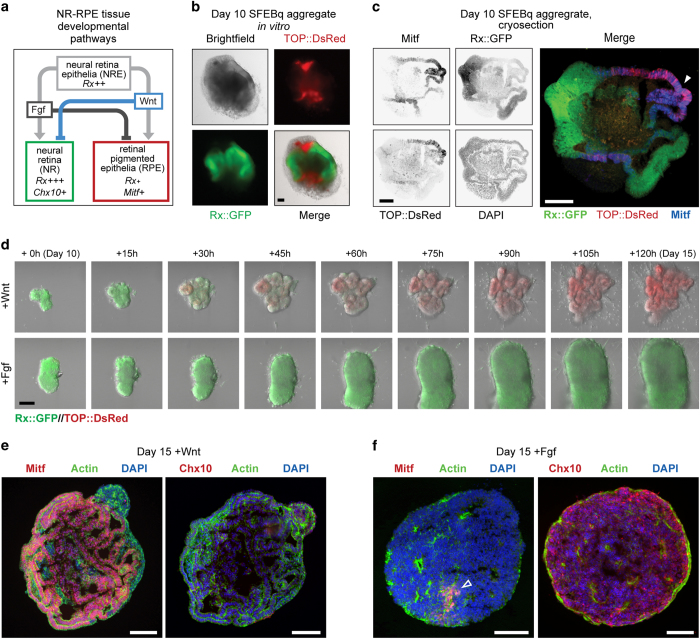
Generation of RPE-like and NR-like tissues *in vitro* using Wnt/β-catenin or Fgf stimulation. (**a**) Schematic of marker gene expression and signaling pathways that promote neural retina epithelial (NRE) tissue to form neural retina (NR) or retinal pigmented epithelial (RPE) tissues. (**b**) Transillumination (Trans) and fluorescent images of a Day 10 SFEBq aggregate generated from Rx::GFP//TOP::DsRed ES cells. The TOP promoter (‘TCF/LEF optimized promoter’)^15^ drives DsRed expression downstream of Wnt/β-catenin signaling. Scale bar 100 μm. (**c**) Immunohistochemistry was performed on cryosections of Day 10 SFEBq Rx::GFP//TOP::DsRed aggregates, closed white arrow showing the overlap of TOP::DsRed and Mitf staining. Scale bar shows 100 μm. (**d**) Montage of images taken from Data Citation 1, showing Day 10 Rx::GFP+//TOP::DsRed tissue in the presence of Wnt/β-catenin (+Wnt) or Fgf stimulation media over 5 days. (**e**,**f**) Immunohistochemistry was performed on Day 15 explants cultured with Wnt-stimulating media (**e**) or Fgf-stimulating media (**f**). Scale bars 100 μm. Wnt stimulation produces aggregates that are majority Mitf+ and Chx10- where as Fgf stimulation produces aggregates that are majority Chx10+ with some aggregates showing small patches of Mitf+ cells (open white arrow).

Both *in vivo* and *in vitro* studies have demonstrated the profound cell fate-promoting effects of Fgf and Wnt/β-catenin signalling pathways during the differentiation of NR and RPE tissues, respectively^1,4,8–14^ (Fig. 2a). Despite these, the transcriptional gene targets of Fgf and Wnt/β-catenin signaling during NR and RPE differentiation have remained incompletely understood, especially at the transcriptome scale.

The principal goal of this study, thus, was to utilize RNA-Seq in combination with SFEBq in order to better understand how Fgf and Wnt/β-catenin signalling affect the transcriptome of Rx+ optic progenitor tissue. Towards this end, we utilized a previously established Rx::GFP reporter mouse ESC line, allowing us to monitor the generation of Rx+ SFEBq tissue in realtime^6,7^.

Using the Rx::GFP reporter line and a Wnt/β-catenin signaling reporter ‘TOP::DsRed’^15^, we confirmed that SFEBq tissue with relatively high Wnt/β-catenin signaling correlated with RPE-like characteristics, such as Mitf expression and a comparatively thin tissue morphology (Fig. 2b,c). Consistently, we found that exposure of Day 10 Rx::GFP+//TOP::DsRed tissue explants to CHIR99201 (a chemical agonist of Wnt/β-catenin signaling^16^, a treatment hereon simply referred to as ‘Wnt stimulation’) strongly activated the TOP::DsRed reporter by Day 12 and resulted in tissue displaying RPE-like morphology by Day 15 (Fig. 2d, Data Citation 1). Conversely, exposing Day 10 Rx::GFP+ tissue explants to Fgf stimulating conditions resulted in highly expressing Rx::GFP+ tissue that displayed NR-like morphology by Day 15 (Fig. 2d, Data Citation 1).

We further analyzed these Day 15 Wnt or Fgf stimulated tissues via immunohistochemistry. Day 15 Wnt stimulated tissue was majority Mitf+, whereas Fgf stimulation produced tissue that was majority Chx10+ (Fig. 2e,f). In addition, we found that Fgf stimulation but not Wnt stimulation allowed the appearance of postmitotic retinal ganglion cells as evidenced by expression of Pou4f2 (also called Brn-3b^17,18^), a marker that was not present in Day 10 Rx::GFP+ tissue (Supplementary Fig. 2a). However, it is important to note that some Fgf stimulated aggregates displayed a small portion of Mitf+ tissue (Fig. 2f), and Wnt stimulated tissue was not 100% positive for Mitf (Fig. 2e). Thus, Wnt and Fgf stimulating conditions produce Day 15 tissue aggregates that are majority, but not absolutely, RPE-like and NR-like in identity.

**Figure 3:**
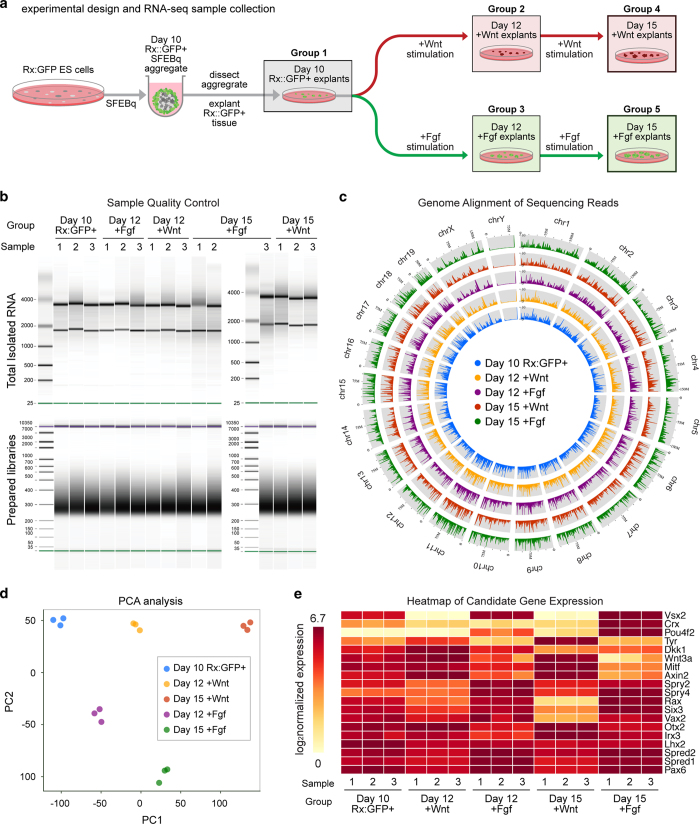
RNA-Seq-based transcriptome analysis of Wnt/β-catenin or Fgf stimulated Day 10 Rx::GFP+ SFEBq tissue. (**a**) Schematic diagram of RNA-Seq experimental design and sample collection. (**b**) Electrophoretic quality control of sample total RNA and prepared libraries using, respectively, the RNA Pico Kit and High Sensitivity DNA Assay Kit using Bioanalyzer (Agilent). Leftmost lanes show marker ladders in base pairs (bp) (**c**) Circos plot showing the genomic coverage of the mapped reads of a sample from each group. (**d**) Global expression profiling using PCA analysis. (**e**) Heatmaps showing the expression of known NRE, RPE, NR, Fgf-target, and Wnt-target genes (see Technical Validation).

We next performed RNA-Seq analyses to measure the gene expression changes in Day 10 Rx::GFP+ tissue following Wnt or Fgf stimulation. We collected five groups of samples in biological triplicate (Fig. 3a): Day 10 Rx::GFP+ tissue (i.e., the starting material, Group 1); Day 12 and Day 15 Fgf stimulated tissue (Day 12 +Fgf and Day 15 +Fgf, Groups 2 and 4); Day 12 and Day 15 Wnt/β-catenin stimulated tissue (Day 12 +Wnt and Day 15 +Wnt, Groups 3 and 5). We then extracted high-quality total RNA from these samples and prepared paired-end libraries for sequencing on an Illumina HiSeq platform (Table 1,Table 2, Fig. 3b). This approach generated on average ~20 million paired-end reads per sample, and all samples possessed a suitable level of read quality and a high mapping rate (Fig. 3c, Technical Validation, Table 3).

Ultimately, this RNA-Seq analysis measured ~18000 gene expression level changes, revealing significant differences in gene expression profiles between the groups (Fig. 3d). In the RNA-Seq data, we examined the expression patterns of some known NR, RPE, Wnt/β-catenin-target, and Fgf-target genes (Fig. 3e, see Technical Validation). Notably, the RNA-Seq data produced gene expression patterns that correlated with the immunohistochemical analyses of Chx10 (Vsx2), Mitf, and Pou4f2 (Supplementary Fig. 2b–f). Nevertheless, it’s important to note that our RPE-like and NR-like tissue samples were generated *in vitro*, and thus, our dataset would not be expected to completely mirror *in vivo* NR and RPE gene expression patterns.

In conclusion, our dataset (GSE62432, Data Citation 2, Supplementary Table 1) is a genetic analysis of how *Rx+* tissue responds to Fgf and Wnt/β-catenin signaling pathways as it differentiates towards NR-like and RPE-like tissue. This data may be helpful for future work in optimizing *in vitro* optic tissue engineering as well as future studies examining the developmental and cellular biology of eye. For instance, with these data, we can ask questions such as:

1. What genes change expression levels following the stimulation of competent Day 10 Rx+ tissue with Wnt/β-catenin or Fgf signalling? (Group 1 versus Group 2, Group 1 versus Group 3).
2. What genes change expression during the maturation stages (Day 12–15) of *in vitro* RPE-like and NR-like tissue differentiation? (Group 2 versus Group 4, Group 3 versus Group 5).
3. What are the major differences in the transcriptome profiles of similarly aged *in vitro* generated RPE-like and NR-like differentiating tissues? (Group 2 versus Group 3, Group 4 versus Group 5)

*In vitro* generated optic tissues have been shown to integrate into host eyes following transplantation and thus hold immense potential in future cell replacement therapies^19–21^. In this regard, our dataset may help facilitate the continued innovation of *in vitro* optic tissue generation techniques and their applications within regenerative medicine.

## Methods

### Generation of NR-like and RPE-like tissues using SFEBq

SFEBq and optic cup culture was performed using Rx::GFP murine ES cells^6^ according to the protocol described by Eiraku and colleagues^22^ with some minor modifications (detailed graphical overview of the SFEBq protocol and optic tissue culture protocol used in this study can be found in Supplementary Fig. 1a–j). For instance, unlike Eiraku and colleagues (2011), we cultured Rx::GFP murine ES cells in the presence of 2i conditioned media (i.e., ES cell media containing 3 μM CHIR99201 and 1 μM PD 0325901) due to its reported effect of promoting a uniform ‘ground state’ within ES cells^23^, and in addition, it had previously been shown that 2i culture improves ES cell differentiation rate of some neuronal lineages^24^. However, because the CHIR99201 compound^16^ induces Wnt/β-catenin signaling via inhibition of GSK3, a property that we later utilized to promote the RPE-like tissue differentiation pathway in Day 10 Rx::GFP+ tissue explants, it was important to confirm that 2i culture of ES cells did not bias ES cells towards a specific tissue fate prior to differentiation experiments. Towards this aim, we used RT-qPCR to examine Day 10 SFEBq aggregates for *rx*, *vsx2*, and *mitf* expression, finding no significant differences between aggregates generated from 2i-cultured or LIF-only cultured ES cells (Supplementary Fig. 2k).

Like Eiraku and colleagues^22^, ES cells were cultured in an ES cell ‘maintenance’ media containing 10% KSR (Gibco, cat. no. 10828–028), LIF (Chemicon, cat. no. ESG1107) and blasticidin (Funakoshi, cat. no. KK-400). To begin SFEBq, ES cells were trypsinized and 3000 cells were reaggregated in 100 μl differentiation media (1.5% KSR) in the wells of a 96-well low-cell-adhesion plate with Lipidure Coat (NOF). Defining trypsinization and reaggregation as Day 1, at Day 2 Matrigel (BD, cat. no. 354230) was introduced to achieve a final Matrigel concentration of 4% (addition of 20 μl of a 24% Matrigel differentiation media solution). This relatively high percentage of Matrigel promotes the formation of a continuous Rx::GFP+ epithelium throughout the periphery of the aggregate versus the sprouting ‘optic cup’ aggregates obtained with lower Matrigel percentages. At Day 10, Rx::GFP+ peripheral tissue was excised using forceps and either collected for RNA-Seq analysis (Group 1) or further cultured in petri dishes containing retinal maturation media (RMM^22^) with either Wnt/β-catenin signaling stimulating conditions (achieved via inhibition of GSK3 using the 3 μM CHIR99201 compound^16^ for the first 24 h and then 1 μM CHIR99201 thereafter) or Fgf-signaling stimulating conditions (5 ng/ml human recombinant bFgf+10% FBS). Media was exchanged at days 11, 12, and 14. RNA-Seq samples were collected in triplicate at Day 10, Day 12 and Day 15. It’s important to note that the RNA-Seq analysis detected endogenous Fgf and Wnt ligands expressed in Day 10 Rx::GFP+ tissue (Supplementary Fig. 1l), and culturing the self-organizing Day 10 Rx::GFP+ tissue without exogenous Wnt of Fgf stimulation (i.e., RMM only) produces Day 15 aggregates that contain a comparatively heterogeneous mix of Mitf+, Chx10+, and Pou4f2+ tissues (Supplementary Fig. 1m). The endogenous expression of Fgf and Wnt ligands in Day 10 Rx::GFP+ tissue means that exogenous Wnt or Fgf stimulation may not completely negate some Fgf or Wnt signaling events in these samples, respectively.

### Immunohistochemistry and live-imaging

Sectioning and immunohistochemistry was performed as previously described^25^. Antibodies were used as follows: Rx: (rabbit, 1:1000, custom^26^, PU42216BS), Chx10 (vsx2): (sheep, 1:1000, Exalpha, X1180P), Mitf: (mouse, 1:1000, Exalpha, X2398M); Pou4f2 (Brn-3b, C-13) (goat, 1:50, Santa Cruz). Actin was visualized with Phalloidin647 (1:2000, Life Technologies). Rx::GFP/TOP::DsRed murine ES cells were generated using a lentivirus. Live-imaging was performed using a glass-bottom dish, Rx::GFP+ tissue from Day 10 Rx::GFP/TOP::DsRed SFEBq aggregates was mounted in Matrigel and filmed using a LCV110 (Olympus) imaging station equipped with 488 and 561 nm excitation lasers.

### RNA extraction, cDNA synthesis, and RT-qPCR

RNA was extracted using the RNeasy kit (Qiagen) using the company-provided protocol. SFEBq aggregates were added to 700 μl buffer RLT and spun through QIAshredder (Qiagen) prior to RNA extraction. The cDNA samples for RT-qPCR reactions were generated using the High Capacity cDNA kit (Applied Biosystems). The qPCR reactions were performed using a 7500 Fast Real-Time PCR System (Applied Biosystems), Taqman Fast Gene Expression Master Mix (Applied Biosystems), and the following TaqMan assays (Applied Biosystems): *gapdh*, Mm99999915_g1; *chx10* (*vsx2*), Mm00432549_m1; *mitf*, Mm00434954_m1; *rx*, Mm01258704_m1. Expression values were calculated using the comparative Ct method with the gene *gapdh* as an internal control. Statistics were performed using Prism (GraphPad Software, Inc.).

### Library preparation and sequencing

Using total RNA extracted as above, sequencing libraries were prepared from 700 ng total so that the library amplification with PCR required no more than 9 cycles. Sequencing was performed on Illumina HiSeq in Rapid mode with 101 cycles, with all the 15 libraries multiplexed in 2 lanes. Details of sequencing and read statistics are described in Table 3. Base calling was processed with RTA 1.17.21.3. Fastq files were generated with bcl2fastq 1.8.4 (illumina) and deposited in the Gene Expression Omnibus (GEO) database under the accession number GSE62432.

### RNA-Seq data analysis

#### Quality check and mapping

The quality of the RNA-Seq reads was evaluated using the version 0.10.1 FastQC quality check package^27^. Having ensured high quality of the data, sequence reads for each library were mapped independently to the mouse genome assembly mm10, using the spliced aligner Tophat (v2.0.8b) with default parameter settings^28^. This yielded a high percentage of unique and properly paired reads, ~87% for all libraries. Next, for each library we estimated the number of sequence reads overlapping at any given nucleotide position in the reference genome at a 100-bp resolution. The count of reads aligning at each position was then normalized to per million reads of their respective library sizes. This data was converted to wiggle-formatted files and eventually used to visualise the coverage of mapped reads in the form of Circos plots. Circos plots were generated using the Circster application of the Galaxy project^29^.

### Expression quantification and downstream analysis

Successfully mapped reads were quantified against the annotated UCSC transcriptome for mm10 to estimate the number of fragments originating from individual genes using the Cuffdiff program of the Cufflinks package^30^ (v2.1.1). The count data estimated by Cuffdiff was then used as the input to bioconductor package edgeR (v3.2.4) to assess the biological variability in the samples and test for differential expression^31^. In addition to identifying the significantly differentially regulated genes edgeR also provides the normalized expression values for each gene in each library. These normalized expression values referred to as counts per million (CPM) were used for all downstream analysis.

Using the normalized expression values we performed Principal Component Analysis (PCA) to assess the variability among the samples as well as the fidelity within the replicates of each sample. Before performing PCA all expression values were log2 transformed and genes with zero values were replaced by the minimum non-zero expression value of the entire dataset. PCA was implemented using the prcomp() function of the R programming language. Furthermore the expression patterns of some of the relevant genes were analysed using the pheatmap package of R. For plotting the heatmap, gene expression values of each gene were normalized by a constant factor representing the highest expression value of that gene across all samples. These values were further scaled up by a factor of 100 and then log-transformed.

## Data Records

Transcriptome-scale expression profile of SFEBq-generated Day 10 Rx::GFP+ optic tissue was performed using RNA-Seq. The Day 10 Rx::GFP+ tissue was stimulated by exogenous Fgf or Wnt signaling culture conditions and profiled at Days 12 and 15 by RNA-Seq. Three biological replicates were provided for all samples for each time point. The raw sequencing data in the form of fastq files and processed data showing normalized expression values has been submitted to Gene Expression Omnibus (GEO). The GEO accession number GSE62432 provides access to all the raw and processed data generated by RNA-Seq (Data Citation 2). The processing of all samples is summarized in Tables 1, 2, 3.

## Technical Validation

### Quality control of RNA, sequencing libraries and high throughput sequencing

Quality of the total RNA was measured by RNA Pico Kit (Agilent) and all samples with sufficiently high RNA Integrity Number (RIN) were used for this study (average RIN was 8.9 with standard deviation of 0.7). Sequencing libraries were evaluated by High Sensitivity DNA Assay Kit (Agilent), which indicated a uniform size range across all libraries (Fig. 3b). Each library was sequenced to a depth of ~20 million reads among which about 87% of the reads mapped uniquely to the mouse genome assembly mm10 (Table 3). In addition PCA plots displayed high agreement between the biological replicates thus ensuring us of a sufficiently high quality dataset (Fig. 3d).

### Phenotypic assessments of RNA-Seq groups

The Day 10—Day 15 RNA-Seq expression patterns for *pou4f2* (Supplementary Fig. 2b), *mitf* (Supplementary Fig. 2c), and *vsx2* (*chx10,* Supplementary Fig. 2f) displayed general agreement with their immunohistochemical analyses shown in Fig. 2e–h *and* Supplementary Fig. 2a. The Day 10—Day 15 RNA-Seq expression patterns for *rax* (Supplementary Fig. 2e) displayed general agreement with the live-imaging analyses shown in Fig. 2d. The pigmented cell marker tyrosinase (*tyr*) also showed upregulation following Wnt stimulation (Supplementary Fig. 2c). Other known NRE, NR, RPE, Wnt-target and Fgf-target genes are displayed in the heatmap found in Fig. 3e. For instance, NRE marker gene *lhx2* decreased as RPE and NR differentiation proceeded (compare Day 10 expression versus Days 12 and 15). Wnt/β-catenin signalling genes and targets^32–34^ *wnt3a*, *axin2*, *dkk1*, *irx3* increased expression following Wnt/β-catenin stimulation. NR expressed genes^1,35,36^ *six3*, *crx*, *vax2*, as well as the *spry* and *spred* Fgf-target genes^37^ displayed increased expression following Fgf-stimulation.

## Usage Notes

For RNA-Seq we recommend using the splice-aware software such as Tophat2 for efficient and accurate mapping to the genome. Expression quantification and differential expression can be best achieved by softwares such as edgeR or DEseq. These programs base their statistical inference on Negative Binomial (NB) distribution, which is required to correctly model the biological variation between samples. Some recent protocols elaborate the details to analyze RNA-Seq data^38,39^.

## Additional Information

**How to cite this article:** Andrabi, M. *et al.* Comparative, transcriptome analysis of self-organizing optic tissues. *Sci. Data* 2:150030 doi: 10.1038/sdata.2015.30 (2015).

## Supplementary Material



Supplementary Table 1

Supplementary Information

## Figures and Tables

**Table 1:** Sample quality and Library preparation.

**Sample**	**Qubit measure (ng/ul)**	**Total RNA QC by Bioanalyzer**		**Library preparation**		**Library QC by Bioanalyzer**
		**Dilution**	**RIN**	**Adapter #**	**# of PCR cycles**	**Average size (bp)**
Day 10 Rx::GFP+, replicate 1	29.0	1/10	8.4	2	8	334
Day 10 Rx::GFP+, replicate 2	38.8	1/20	8.7	4	8	354
Day 10 Rx::GFP+, replicate 3	33.2	1/20	8.7	5	8	339
Day 12 +Fgf, replicate 1	23.6	1/10	10.0	6	8	343
Day 12 +Fgf, replicate 2	32.0	1/20	9.6	7	8	350
Day 12 +Fgf, replicate 3	18.2	1/10	9.2	12	8	334
Day12 +Wnt, replicate 1	56.2	1/20	9.6	13	8	333
Day12 +Wnt, replicate 2	50.6	1/20	9.0	14	9	332
Day12 +Wnt, replicate 3	43.4	1/20	9.6	15	8	349
Day 15 +Fgf, replicate 1	35.2	1/20	7.7	16	9	324
Day 15 +Fgf, replicate 2	34.8	1/20	7.7	18	8	349
Day 15 +Fgf, replicate 3	41.2	1/20	8.4	19	8	351
Day15 +Wnt, replicate 1	24.8	1/20	9.0	3	8	341
Day15 +Wnt, replicate 2	68.6	1/20	9.4	9	9	348
Day15 +Wnt, replicate 3	52.0	1/20	9.9	25	8	350

**Table 2:** RNA-Seq workflow.

**Source**	**Protocol 1**	**Protocol 2**	**Sample Name**	**Protocol 3**	**Data**
Murine Rx::GFP ES cells 1	SFEBq Day 10 Rx::GFP+ explant	RNA extraction	Day10 Rx::GFP, replicate 1	RNA-Seq	GSM1526919
Murine Rx::GFP ES cells 1	SFEBq Day 10 Rx::GFP+ explant	RNA extraction	Day10 Rx::GFP,, replicate 2	RNA-Seq	GSM1526920
Murine Rx::GFP ES cells 1	SFEBq Day 10 Rx::GFP+ explant	RNA extraction	Day10 Rx::GFP,, replicate 3	RNA-Seq	GSM1526921
Murine Rx::GFP ES cells 1	SFEBq Day 10 Rx::GFP+ explant with Fgf treatment until Day 12	RNA extraction	Day 12 +Fgf, replicate 1	RNA-Seq	GSM1526922
Murine Rx::GFP ES cells 1	SFEBq Day 10 Rx::GFP+ explant with Fgf treatment until Day 12	RNA extraction	Day 12 +Fgf, replicate 2	RNA-Seq	GSM1526923
Murine Rx::GFP ES cells 1	SFEBq Day 10 Rx::GFP+ explant with Fgf treatment until Day 12	RNA extraction	Day 12 +Fgf, replicate 3	RNA-Seq	GSM1526924
Murine Rx::GFP ES cells 1	SFEBq Day 10 Rx::GFP+ explant with Wnt treatment until Day 12	RNA extraction	Day12 +Wnt, replicate 1	RNA-Seq	GSM1526925
Murine Rx::GFP ES cells 1	SFEBq Day 10 Rx::GFP+ explant with Wnt treatment until Day 12	RNA extraction	Day12 +Wnt, replicate 2	RNA-Seq	GSM1526926
Murine Rx::GFP ES cells 1	SFEBq Day 10 Rx::GFP+ explant with Wnt treatment until Day 12	RNA extraction	Day12 +Wnt, replicate 3	RNA-Seq	GSM1526927
Murine Rx::GFP ES cells 1	SFEBq Day 10 Rx::GFP+ explant with Fgf treatment until Day 15	RNA extraction	Day 15 +Fgf, replicate 1	RNA-Seq	GSM1526928
Murine Rx::GFP ES cells 1	SFEBq Day 10 Rx::GFP+ explant with Fgf treatment until Day 15	RNA extraction	Day 15 +Fgf, replicate 2	RNA-Seq	GSM1526929
Murine Rx::GFP ES cells 1	SFEBq Day 10 Rx::GFP+ explant with Fgf treatment until Day 15	RNA extraction	Day 15 +Fgf, replicate 3	RNA-Seq	GSM1526930
Murine Rx::GFP ES cells 1	SFEBq Day 10 Rx::GFP+ explant with Wnt treatment until Day 15	RNA extraction	Day15 +Wnt, replicate 1	RNA-Seq	GSM1526931
Murine Rx::GFP ES cells 1	SFEBq Day 10 Rx::GFP+ explant with Wnt treatment until Day 15	RNA extraction	Day15 +Wnt, replicate 2	RNA-Seq	GSM1526932
Murine Rx::GFP ES cells 1	SFEBq Day 10 Rx::GFP+ explant with Wnt treatment until Day 15	RNA extraction	Day15 +Wnt, replicate 3	RNA-Seq	GSM1526933

**Table 3:** Read Statistics.

**Sample**	**Sequencer**	**Run Mode**	**Read Length**	**Total RNA-Seq reads (pairs)**	**Uniquely mapped paired reads**
Day 10 Rx::GFP+, replicate 1	illumina HiSeq 1500	Rapid Run Mode	101 bp paired-end	19,102,239	16,570,924
Day 10 Rx::GFP+, replicate 2	illumina HiSeq 1500	Rapid Run Mode	101 bp paired-end	20,149,842	17,569,762
Day 10 Rx::GFP+, replicate 3	illumina HiSeq 1500	Rapid Run Mode	101 bp paired-end	17,139,161	14,860,821
Day 12 +Fgf, replicate 1	illumina HiSeq 1500	Rapid Run Mode	101 bp paired-end	20,095,528	17,492,925
Day 12 +Fgf, replicate 2	illumina HiSeq 1500	Rapid Run Mode	101 bp paired-end	19,790,146	17,260,416
Day 12 +Fgf, replicate 3	illumina HiSeq 1500	Rapid Run Mode	101 bp paired-end	19,699,644	17,160,000
Day12 +Wnt, replicate 1	illumina HiSeq 1500	Rapid Run Mode	101 bp paired-end	19,550,831	16,885,778
Day12 +Wnt, replicate 2	illumina HiSeq 1500	Rapid Run Mode	101 bp paired-end	17,714,812	15,386,182
Day12 +Wnt, replicate 3	illumina HiSeq 1500	Rapid Run Mode	101 bp paired-end	18,978,467	16,303,935
Day 15 +Fgf, replicate 1	illumina HiSeq 1500	Rapid Run Mode	101 bp paired-end	16,903,783	14,474,402
Day 15 +Fgf, replicate 2	illumina HiSeq 1500	Rapid Run Mode	101 bp paired-end	18,054,446	15,537,681
Day 15 +Fgf, replicate 3	illumina HiSeq 1501	Rapid Run Mode	102 bp paired-end	17,568,261	15,105,314
Day15 +Wnt, replicate 1	illumina HiSeq 1500	Rapid Run Mode	101 bp paired-end	18,951,347	16,373,360
Day15 +Wnt, replicate 2	illumina HiSeq 1500	Rapid Run Mode	101 bp paired-end	15,676,143	13,443,271
Day15 +Wnt, replicate 3	illumina HiSeq 1500	Rapid Run Mode	101 bp paired-end	16,720,389	14,417,934
