# Supplementary material for: Comparative, transcriptome analysis of self-organizing optic tissues
Source: Sci Data. 2015 Jun 23;2:150030. doi: 10.1038/sdata.2015.30 (PMC4477696; doi:10.1038/sdata.2015.30)
Supplement: Supplementary Information [file sdata201530-s3.pdf]

Supplementary Information

Table of Contents:

Page 1 --- Supplementary File Legend

Page 2 --- S.Fig. 1 | Graphical overview of SFEBq protocol, RNA-Seq sample preparation and collection, effect of 2i media on SFEBq optic optic tissue induction, expression of Wn and Fgf ligands in Day 10 Rx::GFP+ tissue explants, and immunostaining of Day 15 explants cultured without exogenous Wnt or Fgf stimulation.

Page 3 --- S.Fig 2 | Immunohistochemical analysis of Pou4f2 and RNA-Seq gene expression profiles of *pou4f2, mitf, tyr, rx,* and *vsx2* (*chx10*).

Supplementary File Legend

Supplementary File 1.xlsx | Searchable spreadsheet of the RNA-Seq data. Triplicate and average expression values (counts per million, cpm) for each group are displayed. Comparisons between the groups (columns Y - AD) are also shown. The default display of the spreadsheet is with cells Day 12 +FGF / Day 12 +Wnt in descending order and with lowly expressed genes omitted. Note, these parameters can be removed by altering the stringency of the filters of columns Y - AD.

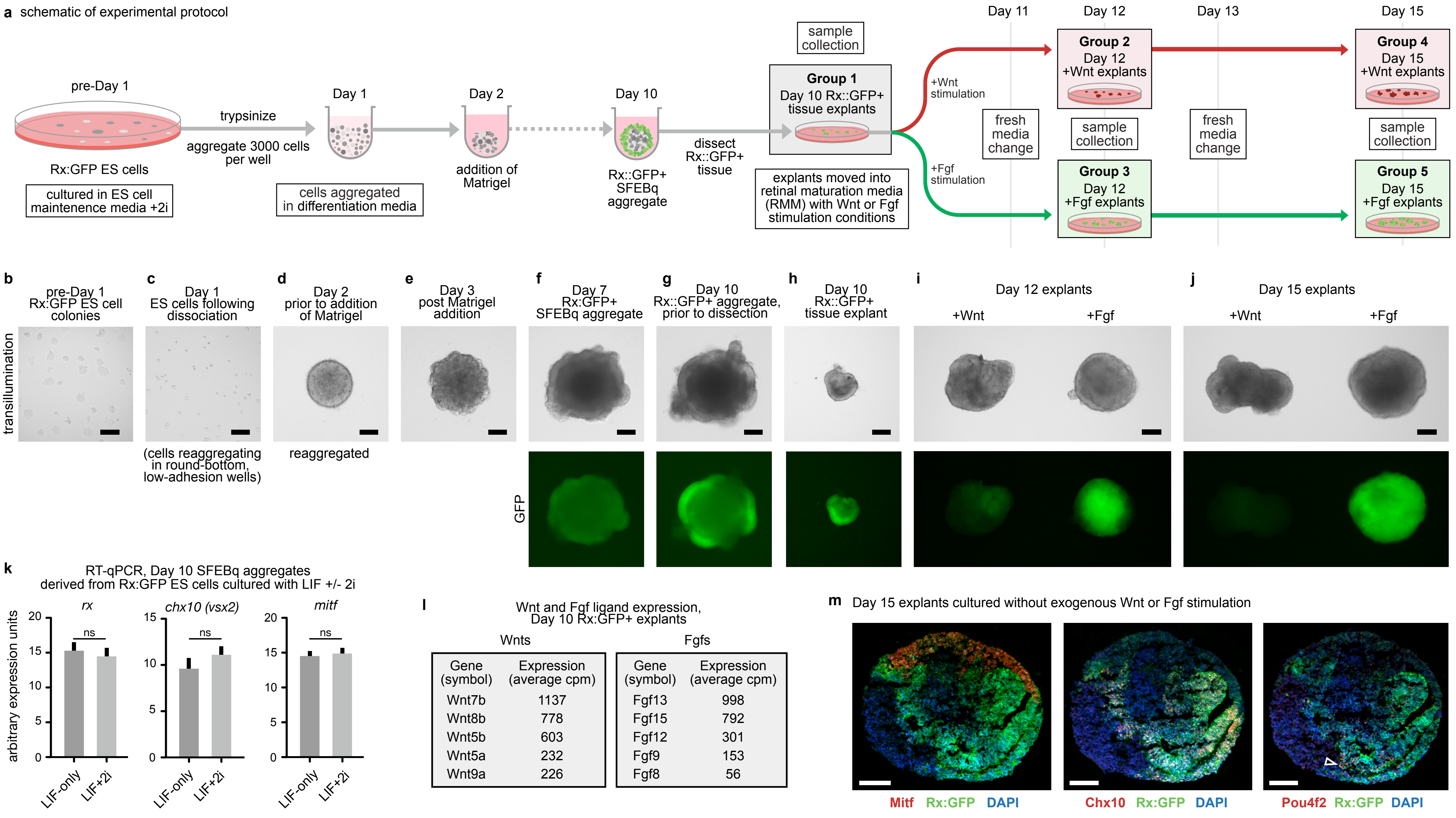

S.Fig. 1 | Graphical overview of SFEBq protocol, RNA-Seq sample preparation and collection, effect of 2i media on SFEBq optic optic tissue induction, expression of Wnt and Fgf ligands in Day 10 Rx::GFP+ tissue explants, and immunostaining of Day 15 explants cultured without exogenous Wnt or Fgf stimulation. **a**, Schematic diagram of SFEBq protocol, sample preparation and RNA-Seq collection. **b**, Transillumination images of representative ES cell colonies prior to dissociation. All scale bars in the figure represent 100 μm. **c**, Transillumination image of dissociated ES cells prior to “reaggregation” in round bottom low-adhesion 96-well plates. **d**, 24h later at Day 2, cells have “reaggregated” at the bottom of the well. Matrigel is then added to a final concentration of 4%. **e**, Following Matrigel addition, the aggregate begins to increase in size as seen in Day 3. **f**, At Day 7, a comparatively faint GFP signal is detectable in the outer layer of the aggregate. **g**, At Day 10, a comparatively thicker and brighter Rx::GFP+ epithelium is present. The aggregate shown is the same aggregate from (f) yet 72 hours older. **h**, Day 10 Rx::GFP+ aggregates are dissected, taking care to remove and isolate the GFP+ peripheral layer. **i**, Rx::GFP+ epithelial explants are cultured in either Wnt or Fgf stimulating conditions, and by Day 12, a difference in Rx::GFP signal is detectable. **j**, At Day 15, the difference in Rx::GFP+ signal between the two conditions remains. **k**, RT-qPCR expression analysis of rx, chx10, mitf expression of Day 10 aggregates derived from ES cells cultured with ES cell media +LIF or ES cell media +LIF +2i. ns, no significance; $P > 0.05$, t-test; n=6 for each group; error bars show standard error of the mean (s.e.m.). **l**, Partial list of Wnt and Fgf ligands detected in Day 10 Rx::GFP+ tissue (Group 1) via RNA-Seq. The five highest expressed Wnt and Fgf ligands are listed. **m**, Immunostainings of Day 10 Rx::GFP+ tissue explants cultured without exogenous Wnt of Fgf stimulation (i.e. RMM media only). These aggregates contain a mixture of Mitf+ RPE-like and Chx10+ NR-like tissue, and also contain postmitotic retinal ganglion cells as recognized by Pou4f2 expression (open white arrow head).

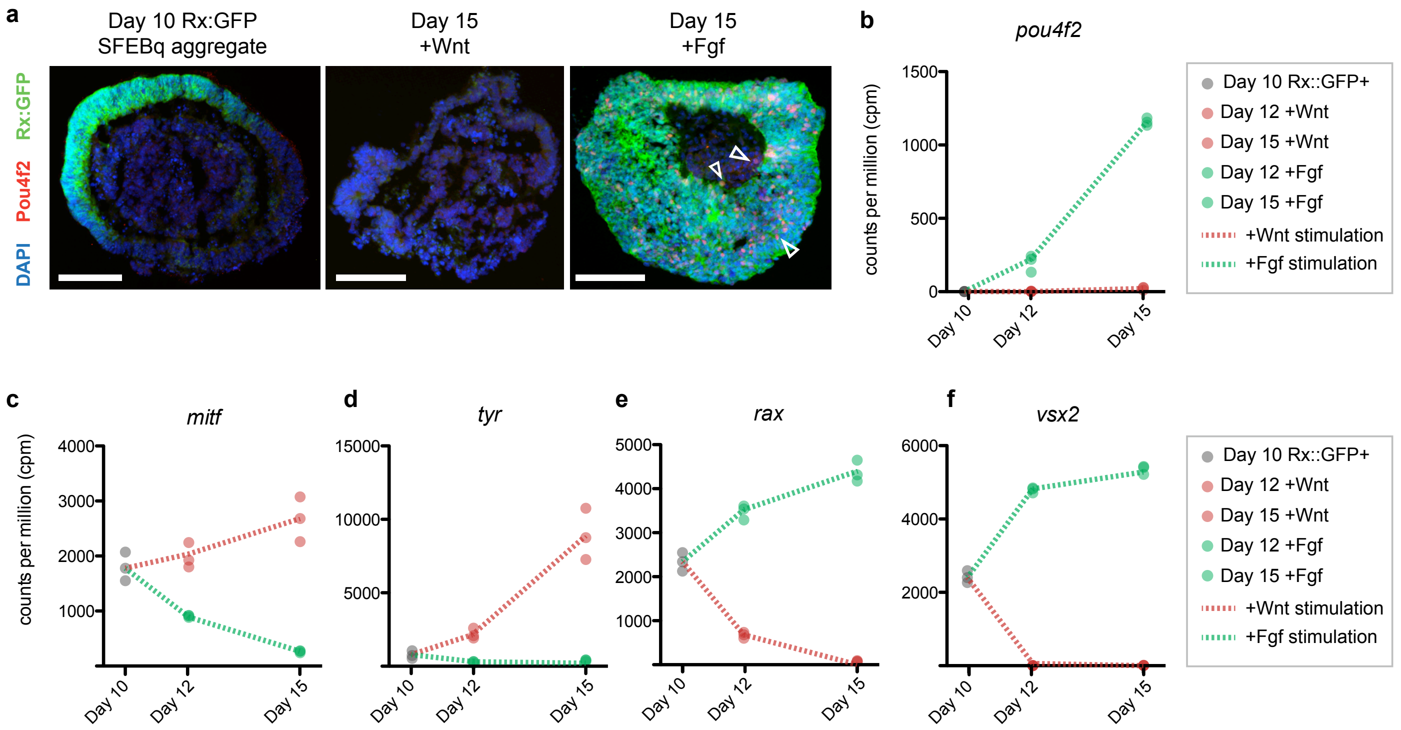

S.Fig 2 | Immunohistochemical analysis of Pou4f2 and RNA-Seq gene expression profiles of *pou4f2, mitf, tyr, rx,* and *vsx2 (chx10)*. a, Immunostainings detecting Pou4f2 in cryosectioned Day 10 Rx::GFP SFEBq, Day 15 +Wnt, and Day 15 +Fgf aggregates. Scale bars represent 100 μm. **b-f**, RNA-Seq expression profiles *pou4f2, mitf, tyr, rx,* and *vsx2 (chx10)*. Triplicates shown at each time point. Connecting lines are for visual references only.
